# Predictive Value of Fibrin Fibrinogen Degradation Products-to-Potassium Ratio for Poor Functional Outcome in Patients with Aneurysmal Subarachnoid Hemorrhage: A Retrospective Case–Control Study

**DOI:** 10.1007/s12028-023-01865-4

**Published:** 2023-10-13

**Authors:** Weida Li, Shuangquan Zhao, Xinlong Chen, Yi Zhang, Ping Lin, Xingyuan Huang, Simeng Yi, Xuehai Deng, Jianlin Ding, Mingkai Xia, Peijun Tang, Xiaoping Tang, Long Zhao

**Affiliations:** 1 Department of Neurosurgery, Affiliated Hospital of North Sichuan Medical College, Nanchong, 637000 China; 2 School of Clinical Medicine, North Sichuan Medical College, Nanchong, 637000 China; 3 Neurosurgical Research Center, Affiliated Hospital of North Sichuan Medical College, Nanchong, 637000 China; 4 Department of Emergency, Affiliated Hospital of North Sichuan Medical College, Nanchong, 637000 China; 5 School of Psychiatry, North Sichuan Medical College, Nanchong, 637000 China; 6 School of Medical Imaging, North Sichuan Medical College, Nanchong, 637000 China

**Keywords:** Aneurysmal subarachnoid hemorrhage, Outcome, Predict, Fibrin fibrinogen degradation products, Potassium, Hypokalemia

## Abstract

**Background:**

The relationship of fibrin(ogen) degradation products (FDPs) and potassium with the functional outcomes of patients with aneurysmal subarachnoid hemorrhage (aSAH) is still uncertain. This study aims to evaluate the predictive value of a novel combination biomarker, the FDP-to-potassium ratio (FPR), for poor functional outcomes in patients with aSAH.

**Methods:**

A total of 425 consecutive patients with aSAH at a single center were retrospectively enrolled in our study. An unfavorable outcome was defined as a modified Rankin Scale (mRS) score of 3–6 at 3 months after discharge. Univariate analysis and multivariable logistic regression were performed for baseline information and laboratory parameters recorded at admission. In addition, the receiver operating characteristic curve was plotted, and propensity score matching was performed based on the FPR.

**Results:**

On the basis of mRS grade, 301 patients were classified as having favorable outcomes, and 124 patients were assessed as having unfavorable outcomes. FPR levels were significantly correlated with mRS grade (*r*[Spearman] = 0.410; *P* < 0.001). Multivariate logistic regression analysis showed that age (odds ratio [OR] 1.043, 95% confidence interval [CI] 1.016–1.071; *P* = 0.002), white blood cell count (OR 1.150, 95% CI 1.044–1.267; *P* = 0.005), potassium (OR 0.526, 95% CI 0.291–0.949; *P* = 0.033), World Federation of Neurosurgical Societies grade (OR 1.276, 95% CI 1.055–1.544; *P* = 0.012), and FPR (OR 1.219, 95% CI 1.102–1.349; *P* < 0.001) at admission were independently associated with poor functional outcomes. The DeLong test showed that the area under the receiver operating characteristic curve of FPR was higher than that of age, white blood cell count, potassium, World Federation of Neurosurgical Societies grade, or FDP alone, indicating that FPR had better predictive potential than these other variables. After 1:1 propensity score matching (FPR ≥ 1.45 vs. FPR < 1.45), the rate of poor prognosis was still significantly increased in the high-FPR group (48/121 [39.7%] vs. 16/121 [13.2%], *P* < 0.001).

**Conclusions:**

Fibrin(ogen) degradation product-to-potassium ratio is an independent predictor of poor outcomes for patients with aSAH and may be a promising tool for clinicians to evaluate patients’ functional prognosis.

**Supplementary Information:**

The online version contains supplementary material available at 10.1007/s12028-023-01865-4.

## Introduction

Aneurysmal subarachnoid hemorrhage (aSAH) is one of the most common causes of stroke worldwide and has higher mortality and morbidity than most neurological diseases [1, 2]. It is generally believed that early brain injury and delayed cerebral ischemia are the main causes of poor prognosis in patients with aSAH, requiring timely identification and intervention [3]. Therefore, timely and accurate prediction of disease progression and functional outcomes is essential for the optimal treatment of patients with aSAH. Many classical scales, such as the World Federation of Neurosurgical Society (WFNS) grade [4] (based on the Glasgow Coma Scale [5]) and the modified Fisher (mFisher) score [6], have been reported to correlate with functional prognosis and are used clinically. However, these scales also have inherent flaws, such as overreliance on clinical manifestations and imaging characteristics [7]. Additionally, the application of clinical scales is restricted in some cases, such as when patients receive sedative drugs and mechanical ventilation [8].

With the rapid advances in aSAH treatment and diagnosis, biomarkers are receiving increasing attention. Biomarkers are usually composed of measurable and quantifiable biological parameters. Compared with traditional scales, biomarkers have the advantage of a superior range of application and are readily quantified. In previous studies, biomarkers have been demonstrated to be promising tools in assessing disease risk and predicting prognosis. For example, serum C-reactive protein and the glucose-phosphate ratio predict prognosis, the platelet-Ca^2+^ ratio and neutrophil-albumin ratio predict complications, and the glucose-potassium ratio is used to assess mortality in patients with aSAH [9–14]. Interestingly, the use of combination biomarkers as risk factors or predictive tools is increasingly reported. Combination biomarkers are usually composed of related and inversely varying parameters in a particular disease; therefore, they are usually more sensitive and representative than single markers [15, 16].

Previous studies have shown that the coagulation and fibrinolytic systems are usually unbalanced for a period after aSAH and are involved in both early brain injury and delayed ischemia [17]. Therefore, biomarkers reflecting coagulation and fibrinolytic activity status may be closely related to disease progression and prognosis. Recently, D-dimer, a fibrinogen degradation product, has been reported as an independent predictor for assessing the complications and prognosis as well as long-term mortality of patients with aSAH [18–22]. However, a similar laboratory parameter, the concentration of fibrin fibrin(ogen) degradation products (FDPs), has not been reported in relation to the prognosis of patients with aSAH. In addition, hypokalemia has been reported to be the most common electrolyte disorder after aSAH and is often combined with other clinical parameters as a predictor of poor prognosis and complications with aSAH [7, 23, 24]. In the present study, we found that FDP is positively associated with unfavorable outcomes of aSAH, whereas potassium showed the opposite association. Therefore, we aimed to explore whether the FDP-to-potassium ratio (FPR) is a valuable combination biomarker for predicting the outcomes of patients with aSAH.

## Methods

### Study Design and Population

**Fig. 1:**
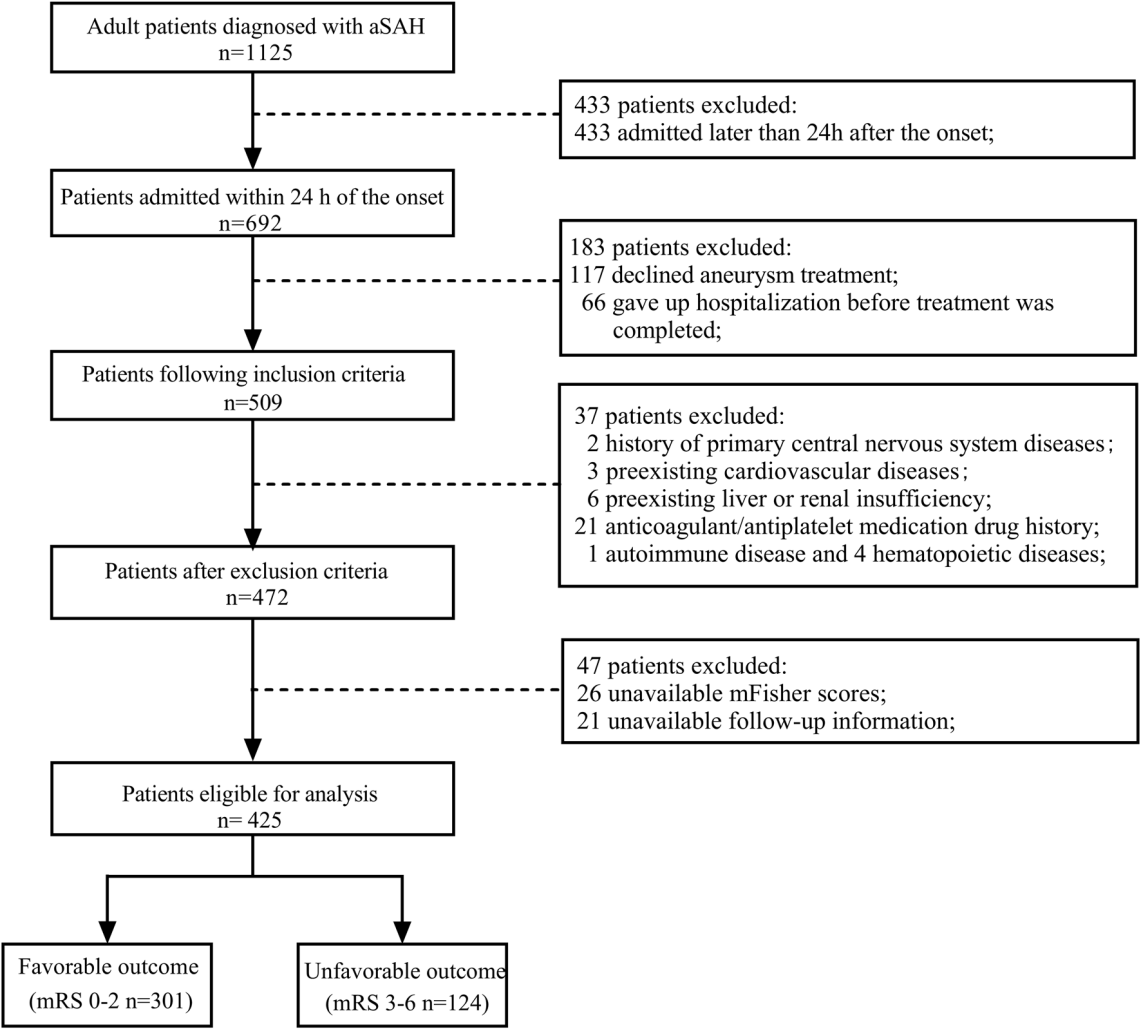
Flow diagram of patients in the study. A total of 425 patients were included in the final study after excluding 700 patients who did not meet the inclusion criteria. We classified the patients according to their 3-month functional outcomes, (mRS 0–2 vs. 3–6). aSAH, aneurysmal subarachnoid hemorrhage, mRS, modified Rankin Scale

The study was performed according to the TRIPOD statement [25] and approved by the Ethical Committee of the Affiliated Hospital of North Sichuan Medical College (No. 2021ER126-1). We retrospectively reviewed the data from consecutive patients with aSAH who were admitted to the Affiliated Hospital of North Sichuan Medical College from August 2016 to June 2022. The inclusion criteria were as follows: (1) age > 18 years; (2) time from onset to admission less than 24 h; (3) aSAH confirmed by digital subtraction angiography or computed tomography angiography; and (4) complete surgical and hospitalization management. The exclusion criteria were as follows: (1) patients gave up hospitalization before treatment was completed; (2) a history of primary or secondary central nervous system diseases; (3) preexisting severe dysfunction of the heart, lung, liver, kidney, and other organs; (4) preexisting autoimmune diseases and hematopoietic diseases; (5) anticoagulant or antiplatelet medication drug history; and (6) insufficient clinical, imaging, laboratory parameters, and follow-up information. The flowchart of study population selection and screening is shown in Fig. 1. We collected all available cases within the specified time window to maximize the power and generalizability of the results. In this series of patients, treatments were terminated for numerous postoperative patients because the guardians were either unable to afford further hospitalization or were pessimistic about the prognosis. We defined this condition as giving up hospitalization before treatment was completed. These patients were excluded due to a possible risk of bias. Because we excluded all cases with insufficient information, this is a complete case analysis.

### Data Collection

Baseline characteristics and clinical information were collected from the electronic database. Baseline characteristics included demographics (age, sex), adverse lifestyle factors (cigarette smoking and alcohol consumption), aneurysm location (anterior circulation, posterior circulation, or both), admission blood pressure (systolic blood pressure, diastolic blood pressure), and medical history (hypertension, diabetes). The WFNS grade was collected as an indicator of disease severity, and the mFisher score was collected to distinguish the radiographic features of SAH. Admission laboratory parameters with possible and reported predictive value based on previous reports were collected; these parameters included white blood cells (WBCs), neutrophils, lymphocytes, red blood cells, hemoglobin, platelets, FDP, potassium, sodium, calcium, glucose, lactate, fibrinogen, activated partial thromboplastin time, thrombin time, and international normalized ratio. The FPR was calculated by dividing the FDP count by the potassium count. For data accuracy and comparability, only the first laboratory parameters after admission were included. We did not extract D-dimer values because most of the included patients were not tested for D-dimer on admission to our hospital.

### Outcome Assessment

The primary outcome was functional outcomes at 3 months after discharge and was evaluated using the modified Rankin Scale (mRS), which has a 7-level scoring system. We defined favorable outcomes as mRS 0 to 2 and unfavorable outcomes as mRS 3 to 6. Outpatient clinic visits and telephone interviews were conducted to assess the mRS grades of patients. All assessments were performed in a blinded manner. Additionally, we analyzed delayed cerebral ischemia (DCI) during hospitalization as the secondary outcome. DCI was defined as the appearance of new clinical features (progressive focal defects, decreased level of consciousness) after all other reasons (severe electrolyte disorder, fever, epilepsy, drug application, hydrocephalus or bleeding, etc.) were ruled out and/or by imaging criteria as new low-density lesions on computed tomography scans or diffusion restriction on diffusion-weighted magnetic resonance imaging [26].

### Statistical Analysis

Statistical analyses were performed using SPSS Statistics 26 (IBM, Armonk, NY) and MedCalc version 18.2.1 (MedCalc Software, Ostend, Belgium), and a two-sided *P* < 0.05 was considered significant. Continuous variables were presented as the mean ± standard deviation or median (interquartile range), whereas categorical variables were presented as counts with percentages (*n* [%]). Normal distribution was assessed by the Shapiro‒Wilk normality test. Continuous variables were compared using Student’s *t* test (normal distribution) or the Mann–Whitney *U*-test (nonnormal distribution). The *χ*^2^ test or Fisher’s exact test was used to compare categorical variables. Spearman’s rank correlation was used for correlation analysis. We performed a multivariable logistic regression analysis to identify independent predictors of functional outcomes, and only variables that had *P* values of less than 0.05 in the univariate analysis were included in the final multivariable model. Additionally, variance inflation factors were calculated to assess multicollinearity and ensure that the variance inflation factors of variables were less than 5 in the final multivariable model.

Furthermore, receiver operating characteristic (ROC) curve analysis was performed to estimate the sensitivity and specificity of each independent predictor, and the area under the curve (AUC) was calculated to measure the prediction ability. DeLong tests were conducted to compare the target variables with other predictors. The patients were divided into a high-FPR group and a low-FPR group according to the best cutoff value achieved by ROC analysis. After propensity score matching (1:1 match, caliper 0.02, nearest neighbor approach) for all possible confounders, the rates of poor outcomes and DCI were compared between the two groups.

## Results

### Baseline Characteristics

After screening according to the inclusion and exclusion criteria, 425 of 1125 adult patients with aSAH were included in this study (Fig. 1). Among the 425 included patients, unfavorable outcomes were documented in 124 patients (29%), and 301 patients (71%) were categorized as having achieved favorable outcomes. There were significant differences in age, WBC, neutrophil, potassium, lactate, FDP, WFNS grade, and mFisher score between the two groups. The differences in the percentage of women, hypertension, diabetes, smoking, alcohol consumption, aneurysm location, and other laboratory data between the groups were not statistically significant (Table 1).

**Table 1:** Univariate analysis of patient characteristics and admission laboratory data associated with functional outcomes at 3 months

Characteristics	All patients (*N* = 425)	90-day functional prognosis		*P* value
		Favorable outcome (*n* = 301)	Unfavorable outcome (*n* = 124)	
Age (year)	61 (52–67)	58 (51–66)	64 (56–70)	** < 0.001**
Female sex, *n* (%)	312 (73.4)	213 (70.8)	99 (79.8)	0.054
BP on admission (mm Hg)				
SBP	147.66 ± 24.37	147.35 ± 24.07	148.41 ± 25.18	0.683
DBP	85 (76–97)	85 (76–95)	87 (75–99)	0.284
Hypertension, *n* (%)	279 (65.6)	193 (64.1)	86 (69.4)	0.302
Diabetes, *n* (%)	19 (4.5)	10 (3.3)	9 (7.3)	0.074
Smoking, *n* (%)	75 (17.6)	54 (17.9)	21 (16.9)	0.805
Alcohol consumption, *n* (%)	61 (14.4)	42 (14.0)	19 (15.3)	0.714
Location of aneurysm, *n* (%)				0.057
Anterior circulation	345 (81.2)	250 (83.0)	95 (76.6)	
Posterior circulation	68 (16.0)	46 (15.3)	22 (17.7)	
Both	12 (2.8)	5 (1.7)	7 (5.7)	
Laboratory data on admission				
WBC (10^9^/L)	10.89 (8.75–13.35)	10.24 (8.56–12.51)	12.71 (9.85–16.29)	** < 0.001**
Neutrophil (10^9^/L)	8.87 (7.08–11.61)	8.68 (6.94–11.19)	9.47 (7.27–12.61)	**0.011**
Lymphocyte (10^9^/L)	0.83 (0.60–1.22)	0.84 (0.61–1.22)	0.81 (0.59–1.18)	0.528
RBC (10^12^/L)	4.07 (3.67–4.46)	4.03 (3.63–4.40)	4.14 (3.72–4.56)	0.078
Hemoglobin (g/L)	121.00 (108.50–132.50)	119.00 (108.00–131.50)	123.50 (110.00–137.00)	0.110
Platelet (10^9^/L)	163.00 (128.00–208.00)	163.00 (130.50–201.50)	163.50 (122.25–215.25)	0.775
Potassium (mmol/L)	3.49 ± 0.45	3.57 ± 0.42	3.31 ± 0.46	** < 0.001**
Sodium (mmol/L)	137.70 (134.95–140.60)	137.40 (134.65–140.35)	138.35 (135.03–141.08)	0.101
Calcium (mmol/L)	2.21 (2.11–2.31)	2.21 (2.11–2.32)	2.21 (2.11–2.28)	0.305
Glucose (mmol/L)	7.27 (6.33–8.90)	7.21 (6.33–8.93)	7.43 (6.31–8.90)	0.664
Lactate (mmol/L)	1.89 (1.43–2.70)	1.89 (1.43–2.70)	2.40 (1.59–3.58)	**0.001**
Coagulation function				
FIB (mg/L)	3.29 (2.76–3.92)	3.23 (2.74–3.82)	3.41 (2.79–4.10)	0.088
APTT (s)	32.00 (29.30–34.70)	32.20 (29.25–34.95)	31.75 (29.53–34.38)	0.471
TT (s)	16.50 (15.70–17.60)	16.60 (15.70–17.60)	16.40 (15.70–17.58)	0.808
INR	1.03 (0.99–1.08)	1.03 (0.99–1.08)	1.04 (0.99–1.08)	0.201
FDP (mg/L)	4.48 (2.94–9.15)	3.90 (2.75–6.03)	9.61 (4.75–19.34)	** < 0.001**
Clinical score on admission				
WFNS grade, *n* (%)				** < 0.001**
1–3	329 (77.4)	257 (85.4)	72 (58.1)	
4–5	96 (22.6)	44 (14.6)	52 (41.9)	
mFisher score, *n* (%)				**0.001**
0–2	136 (32.0)	111 (36.9)	25 (20.2)	
3–4	289 (68.0)	190 (63.1)	99 (79.8)	

Bold figures indicate statistical significance, and values are expressed as *n* (%), mean ± SD, and median (25–75%), respectively

*AC* anterior circulation, *APTT* activated partial thromboplastin time, *BP* blood pressure, *DBP* diastolic blood pressure, *FDP* fibrin(ogen) degradation products, *FIB* fibrinogen, *INR* international normalized ratio, *mFisher* modified Fisher, *PC* post circulation, *RBC* red blood cells, *SBP* systolic blood pressure, *SD* standard deviation, *TT* thrombin time, *WBC* white blood cells, *WFNS* World Federation of Neurosurgical Society

### Correlation Analysis

**Fig. 2:**
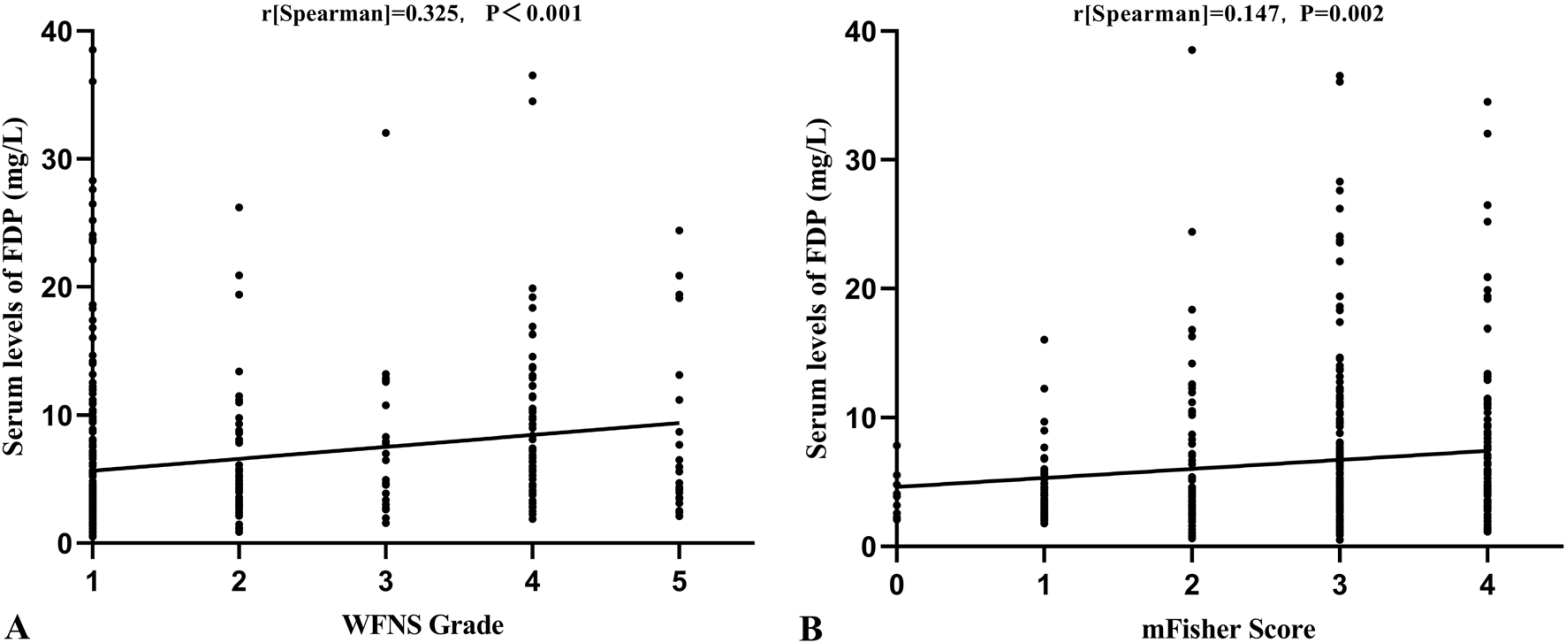
The correlation of FDP with WFNS grade and modified Fisher score. Scatterplot showing that the FDP increased with WFNS grade (Spearman’s *r* = 0.325, *P* < 0.001) and modified Fisher score (Spearman’s *r* = 0.147, *P* = 0.002). FDP, fibrin(ogen) degradation product, mFisher, modified Fisher, WFNS, World Federation of Neurosurgical Society

**Fig. 3:**
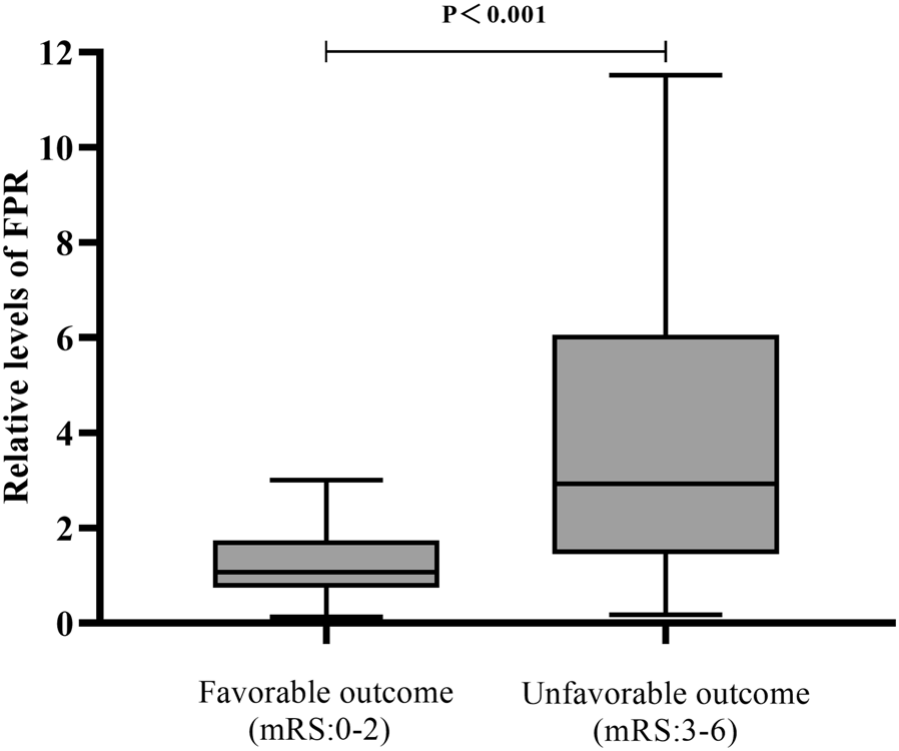
Comparison of FPR between the favorable outcome and unfavorable outcome groups in mRS grade (mRS 0–2 vs. mRS 3–6). Box plots show the median, upper quartiles, and lower quartiles. FPR, fibrin(ogen) degradation products-to-potassium ratio, mRS, modified Rankin Scale

**Fig. 4:**
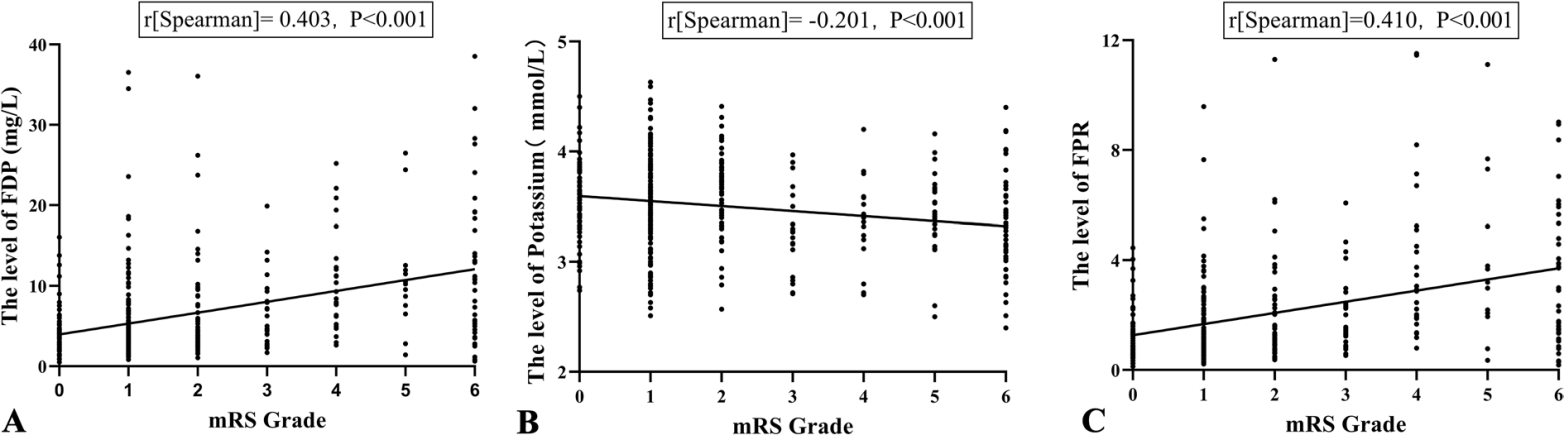
The correlation of FDP, potassium, and FPR levels with mRS grade. Scatterplot showing that the FPR increases with mRS grade (Spearman’s *r* = 0.410, *P* < 0.001). The same trends held with the FDP (Spearman’s *r* = 0.403, *P* < 0.001) and potassium presented a negative correlation trend with increasing mRS grade (Spearman’s *r* =  − 0.201, *P* < 0.001). FDP, fibrin(ogen) degradation products, FPR, fibrin(ogen) degradation products-to-potassium ratio, mRS, modified Rankin Scale

Spearman’s rank correlation analysis showed that FDP was positively correlated with the WFNS grade and mFisher score. The correlation coefficient between FDP and the WFNS grade was 0.325 (*P* < 0.001). The correlation coefficient between FDP and the mFisher score was 0.147 (*P* = 0.002) (Fig. 2). The FPR was markedly increased in patients with unfavorable outcomes compared with those with favorable outcomes (2.93 [1.46–6.06] vs. 1.08 [0.75–1.74], *P* < 0.001, Fig. 3). In the correlation analysis with outcomes (Fig. 4), there was a significant positive correlation between FDP levels and mRS grades (*r*[Spearman] = 0.403; *P* < 0.001); nevertheless, the level of potassium exhibited an opposite trend (*r*[Spearman] =  − 0.201; *P* < 0.001). There was also a significant positive correlation between FPR and mRS grades (*r*[Spearman] = 0.410; *P* < 0.001).

### Predictors of Poor Prognosis

**Fig. 5:**
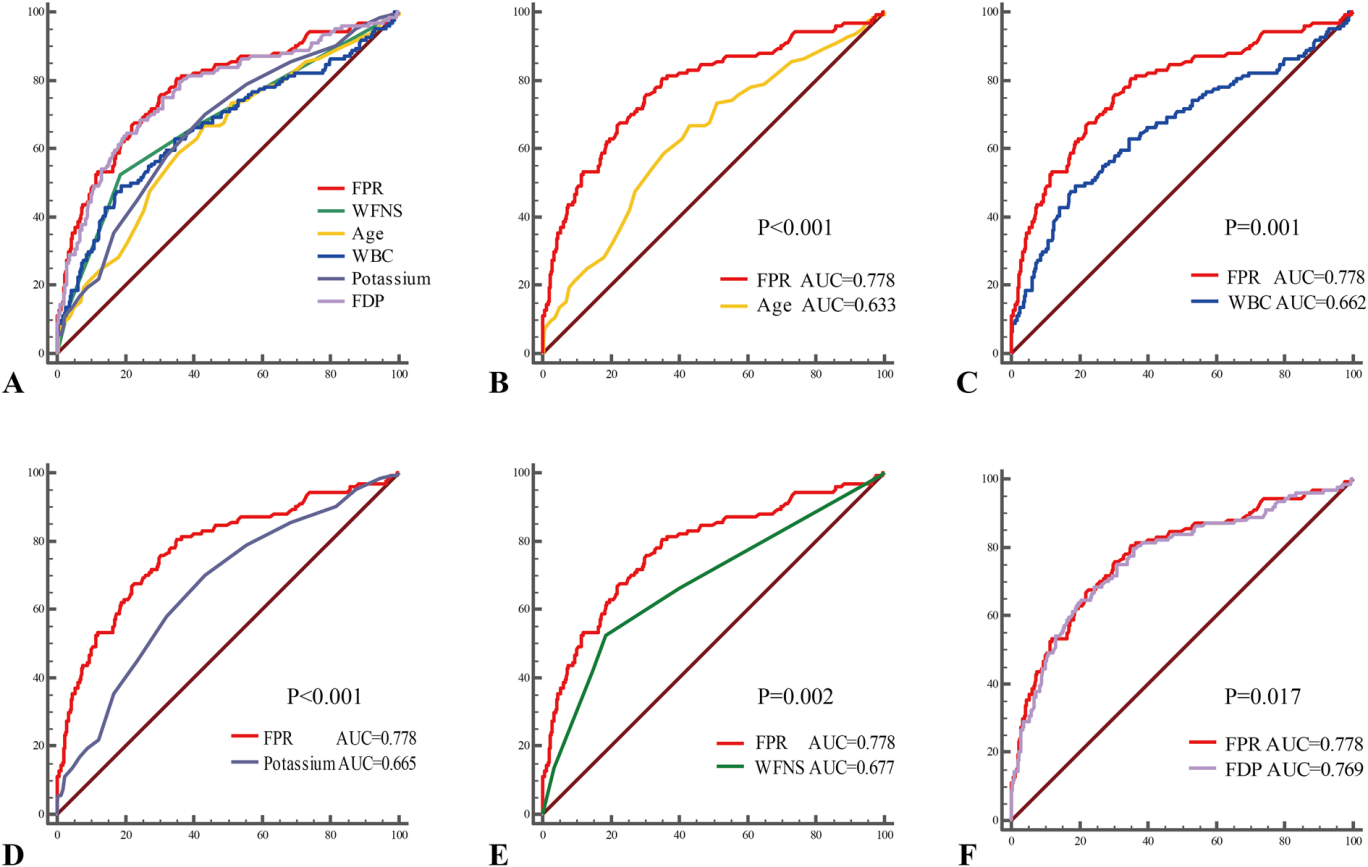
Receiver operating characteristic (ROC) curves for predicting functional outcome at 3 months. Variables include FPR, WFNS grade, age, WBC, potassium, and FDP. Delong tests were performed to compare FPR with other predictors, and results showed the AUC of FPR was significantly higher than that of other predictors. AUC, area under the curve, FDP, fibrin(ogen) degradation products, FPR, fibrin(ogen) degradation products-to-potassium ratio, WBC, white blood cells, WFNS, World Federation of Neurological Society

Multivariable binary logistic regression indicated that FPR (odds ratio [OR] 1.219, 95% confidence interval [95% CI] 1.102–1.349, *P* < 0.001), age (OR 1.043, 95% CI 1.016–1.071, *P* = 0.002), WBC count (OR 1.150, 95% CI 1.044–1.267, *P* = 0.005), potassium (OR 0.526, 95% CI 0.291–0.949, *P* = 0.033), and WFNS grade (OR 1.276, 95% CI 1.055–1.544, *P* = 0.012) were independent predictors of poor functional outcomes (Table 2). Additionally, FDP was not included in the final model after considering multicollinearity. However, in the model that included FDP but not FPR, FDP remained an independent predictor of adverse outcomes (Supplementary Materials Table 1, OR 1.059, 95% CI 1.028–1.090, *P* < 0.001). From the ROC curve analysis, the AUCs of Age, WBC, FPR, FDP, potassium, and WFNS were 0.633 (0.574–0.691), 0.662 (0.601–0.723), 0.778 (0.727–0.830), 0.769 (0.716–0.821), 0.665 (0.608–0.721), and 0.677 (0.617–0.736), respectively (Table 3, Fig. 5a). The AUC of FPR was significantly higher than those of the other predictors (FPR vs. age, *P* < 0.001; FPR vs. WBC, *P* = 0.001; FPR vs. potassium, *P* < 0.001; FPR vs. WFNS, *P* = 0.002; FPR vs. FDP, *P* = 0.017, Fig. 5b–f). The optimal threshold of FPR was identified at 1.45 to predict the poor outcomes of patients with aSAH (Youden’s index = 0.459), in which the sensitivity was 75.81% and the specificity was 70.09% (Table 3).

**Table 2:** Multivariable logistic regression analysis of predictors for poor functional outcome at 3 months

Variables	OR	95% CI	*P* value
Age	1.043	1.016–1.071	**0.002**
WBC	1.150	1.044–1.267	**0.005**
Neutrophil	0.952	0.863–1.051	0.334
FPR	1.219	1.102–1.349	** < 0.001**
Potassium	0.526	0.291–0.949	**0.033**
Lactate	1.138	0.974–1.331	0.103
mFisher score	1.302	0.981–1.728	0.067
WFNS grade	1.276	1.055–1.544	**0.012**

Bold figures indicate statistical significance

*CI* confidence interval, *FPR* fibrin(ogen) degradation products to potassium ratio, *mFisher* modified Fisher, *OR* odds ratio, *WBC* white blood cells, *WFNS* World Federation of Neurosurgical Society

**Table 3:** Area under the curve of baseline patient characteristics associated with poor outcome at 3 months

Variables	Cutoff value	AUC	Sensitivity (%)	Specificity (%)	Youden’s index	95% CI of AUC
Age	60.50	0.633	66.94	56.81	0.238	0.574–0.691
WBC	12.95	0.662	49.19	81.06	0.303	0.601–0.723
FPR	1.45	0.778	75.81	70.09	0.459	0.727–0.830
FDP	6.15	0.769	68.55	75.75	0.443	0.716–0.821
Potassium	3.59	0.665	51.83	76.61	0.284	0.608–0.721
WFNS grade	2.50	0.677	52.42	81.40	0.338	0.617–0.736

*AUC* area under the curve, *CI* confidence interval, *FDP* fibrin(ogen) degradation products, *FPR* fibrin(ogen) degradation products to potassium ratio, *WBC* white blood cells, *WFNS* World Federation of Neurosurgical Society

### Propensity Score Matching

**Fig. 6:**
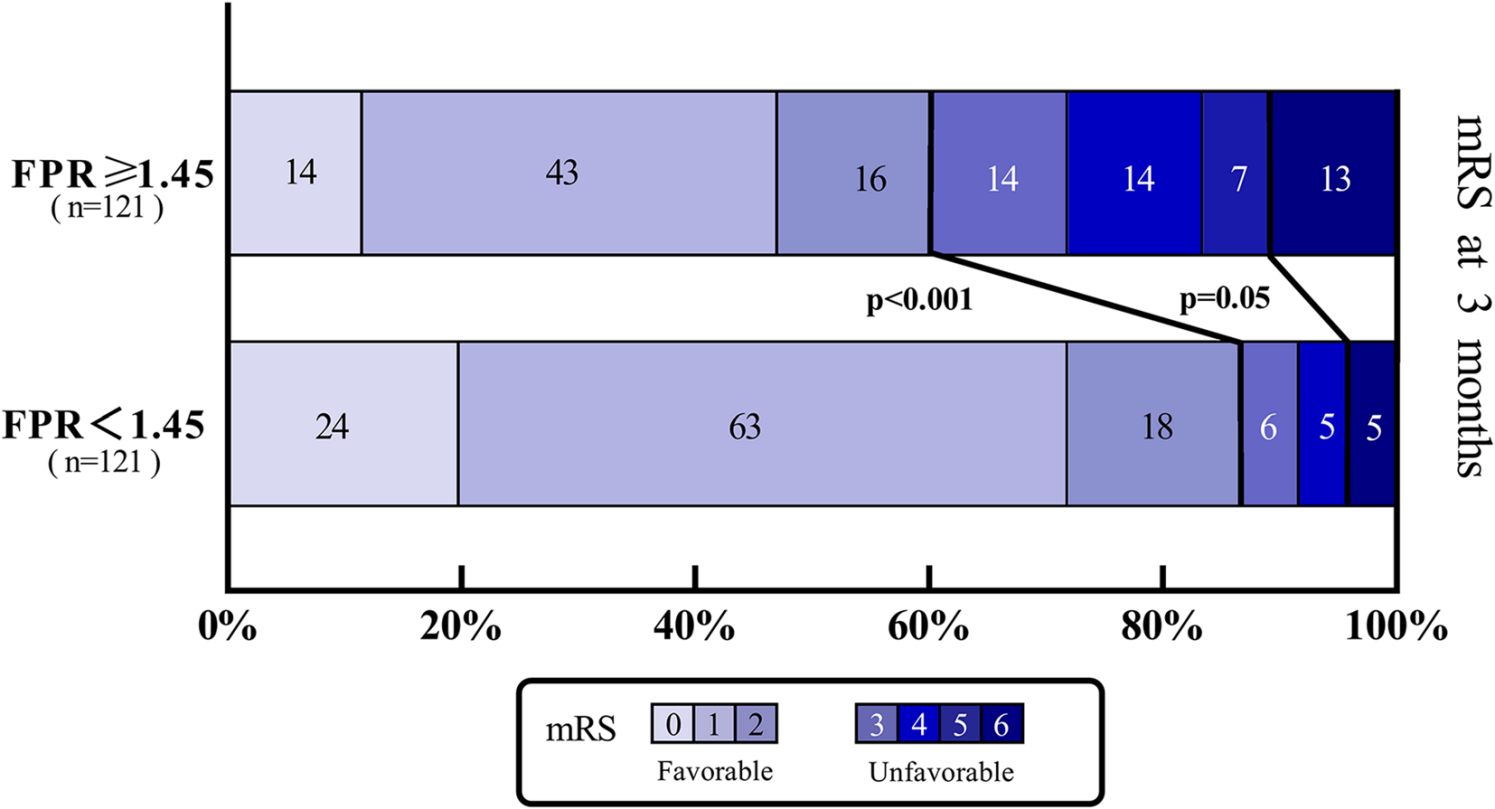
The mRS grade at 3 months for patients with aSAH with FPR level differences (FPR ≥ 1.45 vs. FPR < 1.45) after propensity-score matching. Comparisons of functional outcomes and mortality between the two groups were performed by *χ*^2^ test or Fisher’s exact test. aSAH, aneurysmal subarachnoid hemorrhage, FPR, fibrin(ogen) degradation products-to-potassium ratio, mRS, modified Rankin Scale

The patients were categorized into a high-FPR group (FPR ≥ 1.45 *n* = 186) and a low-FPR group (FPR < 1.45 *n* = 239) based on the optimal threshold. Univariate analysis showed that there were significant differences in age, percentage of women, diastolic blood pressure, smoking, WBCs, neutrophil, lymphocyte, red blood cells, hemoglobin, sodium, calcium, WFNS grade, and mFisher score between the two groups (Table 4). After adjusting for potential confounding with propensity score matching (caliper 0.02, ratio 1:1, nearest neighbor approach), the primary functional outcome and secondary outcome still had statistically significant differences between the two groups (mRS 3–6: FPR ≥ 1.45 48/121 [39.7%] vs. FPR < 1.45 16/121 [13.2%]; *P* < 0.001; DCI: FPR ≥ 1.45 52/121 [43.0%] vs. FPR < 1.45 37/121 [30.6%]; *P* = 0.046). Additionally, the distributions of the mRS grades between the low-FPR and high-FPR groups are shown in Fig. 6. The functional outcomes significantly differed between the high-FPR group and the low-FPR group; however, there was no significant difference in mortality between the two groups at 3 months (FPR ≥ 1.45 13/121 [10.7%] vs. FPR < 1.45 5/121 [4.1%]; *P* = 0.05).

**Table 4:** Characteristics of patients dichotomized to the identified FPR threshold (1.45) before and after PS matching

Characteristics	Before propensity-score matching			After propensity-score matching		
	FPR < 1.45 (*n* = 239)	FPR ≥ 1.45 (*n* = 186)	*P* value	FPR < 1.45 (*n* = 121)	FPR ≥ 1.45 (*n* = 121)	*P* value
Age (year)	60 (51–67)	62 (54–68)	**0.046**	60 (52–68)	61 (52–67)	0.916
Female sex, *n* (%)	165 (69.0)	147 (79.0)	**0.021**	88 (72.7)	86 (71.1)	0.775
BP on admission (mm Hg)						
SBP	145.00 (130.00–162.00)	151.00 (132.00–165.25)	0.080	149.36 ± 25.06	146.74 ± 22.02	0.390
DBP	85.00 (76.00–94.00)	87.00 (76.00–98.25)	**0.048**	86.00 (77.00–95.50)	85.00 (75.50–96.50)	0.831
Hypertension, *n* (%)	150 (62.8)	129 (69.4)	0.156	80 (66.1)	82 (67.8)	0.785
Diabetes, *n* (%)	9 (3.8)	10 (5.4)	0.425	4 (3.3)	5 (4.1)	0.734
Smoking, *n* (%)	53 (22.2)	22 (11.8)	**0.006**	18 (14.9)	17 (14.0)	0.855
Alcohol consumption, *n* (%)	39 (16.3)	22 (11.8)	0.190	17 (14.0)	17 (14.0)	1.000
Location of aneurysm, *n* (%)			0.849			0.688
Anterior circulation	196 (82.0)	149 (80.1)		98 (81.0)	99 (81.8)	
Posterior circulation	37 (15.5)	31 (16.7)		20 (16.5)	17 (14.1)	
Both	6 (2.5)	6 (3.2)		3 (2.5)	5 (4.1)	
Laboratory data on admission						
WBC (10^9^/L)	9.87 (8.46–12.41)	12.02 (9.53–14.54)	** < 0.001**	11.04 (9.04–13.41)	11.09 (8.63–13.05)	0.710
Neutrophil (10^9^/L)	8.58 (6.78–10.82)	9.67 (7.43–12.46)	** < 0.001**	9.72 (8.05–12.41)	9.44 (7.31–11.88)	0.413
Lymphocyte (10^9^/L)	0.86 (0.62–1.27)	0.80 (0.57–1.08)	**0.033**	0.78 (0.56–1.20)	0.76 (0.57–1.06)	0.697
RBC (10^12^/L)	4.12 ± 0.57	3.99 ± 0.66	**0.037**	4.01 ± 0.52	4.02 ± 0.58	0.885
Hemoglobin (g/L)	122.00 (112.00–135.00)	117.00 (105.75–130.00)	**0.006**	118.95 ± 16.40	118.65 ± 19.73	0.899
Platelet (10^9^/L)	168.00 (133.00–207.00)	160.00 (121.00–209.25)	0.452	170.00 (136.50–197.00)	157.00 (118.00–205.50)	0.183
Sodium (mmol/L)	137.30 (134.60–140.20)	138.35 (135.08–141.23)	**0.033**	137.20 (135.00–140.10)	138.00 (134.90–140.60)	0.454
Calcium (mmol/L)	2.23 (2.14–2.2.33)	2.19 (2.08–2.27)	**0.002**	2.20 ± 0.13	2.20 ± 0.15	0.978
Glucose (mmol/L)	7.21 (6.21–8.60)	7.43 (6.50–9.20)	0.081	7.60 (6.35–9.01)	7.24 (6.41–9.10)	0.830
Lactate (mmol/L)	1.89 (1.44–2.76)	2.11 (1.52–3.08)	0.077	2.02 (1.41–2.87)	1.93 (1.40–2.96)	0.690
Coagulation function						
FIB (mg/L)	3.27 (2.77–3.82)	3.32 (2.74–4.08)	0.636	3.17 (2.73–3.90)	3.30 (2.67–3.94)	0.781
APTT (s)	31.90 (29.40–35.10)	32.10 (29.30–34.53)	0.559	31.80 (29.35–34.45)	32.30 (29.10–34.65)	0.944
TT (s)	16.50 (15.70–17.50)	16.50 (15.70–17.60)	0.946	16.50 (15.55–17.55)	16.60 (15.75–17.60)	0.768
INR	1.03 (0.99–1.08)	1.03 (0.98–1.09)	0.526	1.03 (1.00–1.09)	1.04 (0.98–1.10)	0.930
Clinical score on admission						
WFNS grade, *n* (%)			** < 0.001**			0.631
1–3	212 (88.7)	117 (62.9)		98 (81.0)	95 (78.5)	
4–5	27 (11.3)	69 (37.1)		23 (19.0)	26 (21.5)	
mFisher score, *n* (%)			** < 0.001**			0.395
0–2	92 (38.5)	44 (23.7)		38 (31.4)	32 (26.4)	
3–4	147 (61.5)	142 (76.3)		83 (68.6)	89 (73.6)	
Primary outcome						
mRS grade, *n* (%)			** < 0.001**			** < 0.001**
0–2	209 (87.4)	92 (49.5)		105 (86.8)	73 (60.3)	
3–6	30 (12.6)	94 (50.5)		16 (13.2)	48 (39.7)	
Secondary outcome						
DCI, *n* (%)	58 (24.3)	86 (46.2)	** < 0.001**	37 (30.6)	52 (43.0)	**0.046**

Bold figures indicate statistical significance and values are expressed as *n* (%), mean ± SD, and median (25%–75%) respectively.  Propensity-score matching (caliper 0.02, ratio 1:1, nearest neighbor approach) was performed according to the following parameters: age, female sex, DBP, smoking, WBC, neutrophil, lymphocyte, RBC, hemoglobin, sodium, calcium, WFNS grade, and mFisher score

*AC* anterior circulation, *APTT* activated partial thromboplastin time, *BP* blood pressure, *DBP* diastolic blood pressure, *DCI* delayed cerebral ischemia, *FDP* fibrin(ogen) degradation products, *FIB* fibrinogen, *FPR* fibrin(ogen) degradation products to potassium ratio, *INR* international normalized ratio, *mFisher* modified fisher, *PC* post circulation, *PS*, xxx, *RBC* red blood cells, *SBP* systolic blood pressure, *SD* standard deviation, *TT* thrombin time, *WBC* white blood cells, *WFNS*, World Federation of Neurosurgical Society

## Discussion

In this retrospective study, we investigated the association of clotting-related biomarkers with the functional outcome of patients with aSAH. A significant correlation between elevated FDP and FPR levels and unfavorable outcomes (mRS 3–6) was observed. In addition, multivariable regression analyses and propensity score matching confirmed that FPR was an independent predictor of functional outcomes in patients with aSAH. Therefore, we considered serum FPR to be a valuable predictor of aSAH prognosis. To our knowledge, our study is the first to explore the association between FPR and functional outcomes in patients with aSAH.

The results of this study provide new independent and composite biomarkers for predicting clinical outcomes in patients with aSAH. Compared with the clinical grading scale, biomarkers offer the following advantages. First, the use of biomarkers to predict prognosis relies on objective laboratory parameters and reduces the subjective bias caused by manual assessment. Second, biomarkers can be easily measured by routine equipment. This means that a broader scope of application, including conditions such as sedative drugs and mechanical ventilation, compensates for the inherent flaws of the clinical grading scale. Third, evaluating patient outcomes by biomarkers differs fundamentally from grading scales and offers novel options to recognize critical information on the prognosis of patients.

Following the fact that D-dimer has been reported as an independent predictor of poor prognosis and death in aSAH [18–22], our results indicated that FDP was positively correlated with admission WFNS grade and 3-month mRS in patients with aSAH, suggesting that patients with higher FDP values were more likely to have more severe clinical manifestations and worse prognosis. FDP serves as an indicator of the clotting system fibrinolytic system and mainly reflects the production or degradation of its precursor fibrin(ogen) in the acute phase. Fibrinogen is an acute phase response pleiotropic protein synthesized in the liver and plays a crucial role in coagulation, inflammation, and tissue repair [27, 28]. In general, fibrinogen circulates as a soluble homodimer form in the blood. When the coagulation cascade is activated, thrombin converts fibrinogen into insoluble fibrin to participate in thrombosis. Finally, fibrin clots degrade into soluble FDP by plasmin, and the significantly increased level of FDP suggests organisms in the hypercoagulative state and hyperfibrinolysis symptoms [29, 30]. Previous studies have reported that FDP can predict neurological outcomes for patients with cardiac arrest and is associated with the recurrence of chronic subdural hematoma [31, 32]. When aSAH occurs, the integrity of vascular endothelial cells and the blood‒brain barrier are disrupted, leading to secondary coagulation abnormalities and excessive activation of the fibrinolytic system, producing a significant amount of FDPs. Fibrin and fibrinogen are also involved in the onset and progression of neuroinflammation [33, 34]. Fibrin induces neuroinflammation through microglia activation and recruitment of peripheral inflammatory macrophages into the central nervous system. The neuroinflammatory functions of fibrinogen are mainly mediated by the activation of microglia, resulting in neurological deficits [29, 35, 36]. A large amount of FDP suggests excessive consumption of fibrin(ogen), indicating intense neuroinflammation after the onset of aSAH. In addition, fibrin(ogen) is associated with neurodegenerative disorders by promoting *β*-amyloid deposition, inducing astrocytes to form glial scars, and inhibiting axonal growth following aSAH. All of these findings support the conjecture that FDP may be related to the prognosis of aSAH. To date, there have been no reports on the relationship between FDP and the prognosis of patients with aSAH. However, because of the lack of D-dimer data, we could not compare the predictive value of FDP and D-dimer for the prognosis of aSAH. Although it has been reported that D-dimer is more specific and stable than FDP, their clinical value in aSAH needs to be further studied and compared [37].

The potassium cation is of primary physiological importance in maintaining the acid‒base balance and regulating osmotic pressure after aSAH [23]. Our research found that patients with unfavorable outcomes were more likely to develop hypokalemia and that potassium levels were negatively associated with 3-month prognosis. This phenomenon was also reported by Jung et al. and Zhang et al. in a previous study [9, 14]. The reason that patients with aSAH have a lower serum potassium level is due to excessive secretion of catecholamines and *β*^2^ adrenergic hormones, which induce overactivation of the Na^+^/K^+^-ATPase pump as well as increased influx of potassium [38–40]. Additionally, a large amount of potassium deposits in the brain extracellular space because of blood‒brain barrier disruption may be associated with the inversion of neurovascular coupling and cause dysfunction of the nervous system [41–43]. Furthermore, patients with aSAH often lose abundant potassium from the digestive tract because of vomiting due to increased intracranial pressure. Ultimately, these factors collectively promote the occurrence of hypokalemia in patients with aSAH.

Because FDP and potassium levels were positively and negatively correlated with mRS grades in patients with aSAH, we verified the predictive value of the FDP/potassium ratio for functional outcomes. Our results show not only that FPR is independently associated with outcome in patients with aSAH but also that its AUC is significantly higher than those of other independent predictors such as age, WFNS grade, WBCs, and potassium and FDP alone. These results indicate that FPR is an ideal predictor of prognosis in patients with aSAH. Although there is no proven pathophysiological evidence for the relationship between FDP and potassium, they may have an indirect correlation in patients with aSAH. We propose the following conjectures to explain the correlation between FDP and potassium. When aSAH occurs, inflammatory responses and coagulation mechanisms are activated, which cause elevated fibrin(ogen) and correlative FDP. In the acute phase, much fibrin and fibrinogen are rapidly synthesized in the liver. This consumes significant amounts of energy and substrate. Potassium, as the common substrate of protein synthesis and energy generation, is heavily consumed during this period. In addition, catecholamines and *β*^2^ adrenergic hormones are abundantly released, directly promoting potassium influx and fibrin(ogen) synthesis. Regarding the relationship between FDP and potassium, we propose the following conjectures. When aSAH occurs, inflammatory responses and coagulation mechanisms are activated, which cause elevated fibrin(ogen) and correlative FDP. In the acute phase, large amounts of fibrin and fibrinogen are rapidly synthesized in the liver. This consumes significant amounts of energy and substrate. Potassium, as the common substrate of protein synthesis and energy generation, is heavily consumed during this period. In the meantime, catecholamines and *β*^2^ adrenergic hormones are abundantly released, directly promoting potassium influx and fibrin(ogen) synthesis.

Finally, we must stress that although biomarkers have some advantages over grading scales, the function of the grading scale cannot be completely abolished by biomarkers in clinical practice. In this study, although FPR was independently associated with poor prognosis and showed a higher AUC and sensitivity, the specificity of FPR was lower than that of WBC count and WFNS grade. In addition, in our data, we noted that 30 patients (12.6%) with FPR below the threshold had a poor prognosis, while 92 patients (49.5%) with FPR above the threshold had a good prognosis. This shows the limitation of FPR alone in predicting prognosis because other factors, such as age and WFNS grade, are also independently associated with poor prognosis and have independent predictive value. Therefore, we believe that establishing a comprehensive predictive model including FPR, WFNS grade, age, and WBC count in the future will improve the prediction efficiency and provide better clinical guidance.

## Limitations of the Study

We must admit that some limitations exist in this study. First, this was a single-center, retrospective study. Therefore, there may exist unavoidable bias that influences the accuracy of the conclusions. We need a multicenter, prospective study with a large sample size to validate our conclusions. Second, we may have overlooked the influence of some factors, such as nutritional status and financial hardship, on functional outcome. Third, not all laboratory parameters were completely included in our study due to limitations of research conditions, such as D-dimer and C-reactive protein. Finally, because of insufficient data, we merely analyzed the relationship of admission FDP and FPR with prognosis and did not consider the trends in FDP and FPR, which may have caused us to underestimate the role of FDP and FPR in predicting the prognosis of patients with aSAH.

## Conclusions

Our research demonstrated that elevated serum FPR levels were related to poor functional outcomes in patients with aSAH at 3 months and that FPR was an independent predictor of poor prognosis. FPR may be a promising biomarker for predicting the prognosis of patients with aSAH.

### Supplementary Information

Below is the link to the electronic supplementary material.

Supplementary file1 (DOCX 17 KB)

## References

[1] Suwatcharangkoon S, Meyers E, Falo C (2016). Loss of consciousness at onset of subarachnoid hemorrhage as an important marker of early brain injury. JAMA Neurol.

[2] Suarez JI, Tarr RW, Selman WR (2006). Aneurysmal subarachnoid hemorrhage. N Engl J Med.

[3] van Lieshout JH, Dibué-Adjei M, Cornelius JF (2018). An introduction to the pathophysiology of aneurysmal subarachnoid hemorrhage. Neurosurg Rev.

[4] Teasdale GM, Drake CG, Hunt W (1988). A universal subarachnoid hemorrhage scale: report of a committee of the World Federation of Neurosurgical Societies. J Neurol Neuros Psychiatry.

[5] Teasdale G, Jennett B (1974). Assessment of coma and impaired consciousness. A practical scale. Lancet (Lond Engl).

[6] Frontera JA, Claassen J, Schmidt JM, et al. Prediction of symptomatic vasospasm after subarachnoid hemorrhage: the modified fisher scale. Neurosurgery 2006;59(1):21–7; discussion 21–7. 10.1227/01.Neu.0000218821.34014.1b. 10.1227/01.neu.0000243277.86222.6c 16823296

[7] Fujiki Y, Matano F, Mizunari T (2018). Serum glucose/potassium ratio as a clinical risk factor for aneurysmal subarachnoid hemorrhage. J Neurosurg.

[8] Fung C, Inglin F, Murek M (2016). Reconsidering the logic of World Federation of Neurosurgical Societies grading in patients with severe subarachnoid hemorrhage. J Neurosurg.

[9] Zhang D, Zhuang Z, Wei Y (2019). Association of admission serum glucose-phosphate ratio with severity and prognosis of aneurysmal subarachnoid hemorrhage. World Neurosurg.

[10] Qiu SZ, Zheng GR, Chen B, Huang JJ, Shen J, Mao W (2020). Prognostic value of admission serum glucose-phosphate ratio in predicting the 6-month outcome of patients with severe traumatic brain injury: a retrospective study. Clin Chim Acta.

[11] Fountas KN, Tasiou A, Kapsalaki EZ (2009). Serum and cerebrospinal fluid C-reactive protein levels as predictors of vasospasm in aneurysmal subarachnoid hemorrhage. Clin Artic Neurosurg Focus.

[12] Wang XT, Xu YH (2022). Platelets-to-serum Ca2+ ratio as a risk factor for postoperative cerebral vasospasm in surgically treated aneurysmal subarachnoid hemorrhage patients. Eur Rev Med Pharmacol Sci.

[13] Zhang X, Zhang S, Wang C, Liu R, Li A (2022). High neutrophil-to-albumin ratio predicts postoperative pneumonia in aneurysmal subarachnoid hemorrhage. Front Neurol.

[14] Jung HM, Paik JH, Kim SY, Hong DY (2021). Association of plasma glucose to potassium ratio and mortality after aneurysmal subarachnoid hemorrhage. Front Neurol.

[15] He J, Zhang Y, Li T (2023). Glucose-albumin ratio as new biomarker for predicting mortality after intracerebral hemorrhage. Neurosurg Rev.

[16] Navarro J, Kang I, Hwang HK, Yoon DS, Lee WJ, Kang CM (2019). Glucose to lymphocyte ratio as a prognostic marker in patients with resected pT2 gallbladder cancer. J Surg Res.

[17] Geraghty JR, Testai FD (2017). Delayed cerebral ischemia after subarachnoid hemorrhage: beyond vasospasm and towards a multifactorial pathophysiology. Curr Atheroscler Rep.

[18] Fang F, Wang P, Yao W (2022). Association between D-dimer levels and long-term mortality in patients with aneurysmal subarachnoid hemorrhage. Neurosurg Focus.

[19] Fukuda H, Lo B, Yamamoto Y (2017). Plasma D-dimer may predict poor functional outcomes through systemic complications after aneurysmal subarachnoid hemorrhage. J Neurosurg.

[20] Hurth H, Birkenhauer U, Steiner J, Schlak D, Hennersdorf F, Ebner FH (2020). Delayed cerebral ischemia in patients with aneurysmal subarachnoid hemorrhage—serum D-dimer and C-reactive protein as early markers. J Stroke Cerebrovasc Dis.

[21] Juvela S, Siironen J (2006). D-dimer as an independent predictor for poor outcome after aneurysmal subarachnoid hemorrhage. Stroke.

[22] Liu JH, Li XK, Chen ZB (2017). D-dimer may predict poor outcomes in patients with aneurysmal subarachnoid hemorrhage: a retrospective study. Neural Regener Res.

[23] Chen I, Mitchell P (2016). Serum potassium and sodium levels after subarachnoid haemorrhage. Br J Neurosurg.

[24] Wang J, Feng Q, Zhang Y, Qiu W, Gao H (2021). Elevated glucose-potassium ratio predicts preoperative rebleeding in patients with aneurysmal subarachnoid hemorrhage. Front Neurol.

[25] Collins GS, Reitsma JB, Altman DG, Moons KG (2015). Transparent reporting of a multivariable prediction model for individual prognosis or diagnosis (TRIPOD): the TRIPOD statement. BMJ (Clin Res Ed).

[26] Vergouwen MD, Vermeulen M, van Gijn J (2010). Definition of delayed cerebral ischemia after aneurysmal subarachnoid hemorrhage as an outcome event in clinical trials and observational studies: proposal of a multidisciplinary research group. Stroke.

[27] Vilar R, Fish RJ, Casini A, Neerman-Arbez M (2020). Fibrin(ogen) in human disease: both friend and foe. Haematologica.

[28] Davalos D, Akassoglou K (2012). Fibrinogen as a key regulator of inflammation in disease. Semin Immunopathol.

[29] Petersen MA, Ryu JK, Akassoglou K (2018). Fibrinogen in neurological diseases: mechanisms, imaging and therapeutics. Nat Rev Neurosci.

[30] Göbel K, Eichler S, Wiendl H, Chavakis T, Kleinschnitz C, Meuth SG (2018). The coagulation factors fibrinogen, thrombin, and factor XII in inflammatory disorders—a systematic review. Front Immunol.

[31] Ono Y, Hayakawa M, Maekawa K (2017). Fibrin/fibrinogen degradation products (FDP) at hospital admission predict neurological outcomes in out-of-hospital cardiac arrest patients. Resuscitation.

[32] Hori YS, Ebisudani Y, Aoi M, Fukuhara T (2018). Elevated serum fibrinogen degradation products on admission is a novel predictive factor for recurrence of chronic subdural hematoma. World Neurosurg.

[33] Yakovlev S, Strickland DK, Medved L (2022). Current view on the molecular mechanisms underlying fibrin(ogen)-dependent inflammation. Thromb Haemost.

[34] Luyendyk JP, Schoenecker JG, Flick MJ (2019). The multifaceted role of fibrinogen in tissue injury and inflammation. Blood.

[35] Norris EH, Strickland S (2017). Fibrinogen in the nervous system: glia beware. Neuron.

[36] Ryu JK, Davalos D, Akassoglou K (2009). Fibrinogen signal transduction in the nervous system. J Thromb Haemost JTH.

[37] Tsantes AG, Parastatidou S, Tsantes EA (2023). Sepsis-induced coagulopathy: an update on pathophysiology, biomarkers, and current guidelines. Life (Basel, Switzerland).

[38] Chen S, Li Q, Wu H, Krafft PR, Wang Z, Zhang JH (2014). The harmful effects of subarachnoid hemorrhage on extracerebral organs. BioMed Res Int.

[39] Massara F, Tripodina A, Rotunno M (1970). Propranolol block of epinephrine-induced hypokaliaemia in man. Eur J Pharmacol.

[40] Beseoglu K, Etminan N, Steiger HJ, Hänggi D (2014). The relation of early hypernatremia with clinical outcome in patients suffering from aneurysmal subarachnoid hemorrhage. Clin Neurol Neurosurg.

[41] Koide M, Bonev AD, Nelson MT, Wellman GC (2012). Inversion of neurovascular coupling by subarachnoid blood depends on large-conductance Ca2+-activated K+ (BK) channels. Proc Natl Acad Sci USA.

[42] Antunes AP, Schiefecker AJ, Beer R (2014). Higher brain extracellular potassium is associated with brain metabolic distress and poor outcome after aneurysmal subarachnoid hemorrhage. Crit Care (Lond Engl).

[43] Ybanez N, Agrawal V, Tranmer BI, Gennari FJ (2014). Severe hypokalemia in a patient with subarachnoid hemorrhage. Am J Kidney Dis.

