# Supplementary material for: Predictive Value of Fibrin Fibrinogen Degradation Products-to-Potassium Ratio for Poor Functional Outcome in Patients with Aneurysmal Subarachnoid Hemorrhage: A Retrospective Case–Control Study
Source: Neurocrit Care. 2023 Oct 13;40(3):1013–24. doi: 10.1007/s12028-023-01865-4 (PMC11147889; doi:10.1007/s12028-023-01865-4)
Supplement: Supplementary file 1 — Supplementary file1 (DOCX 17 KB) [file 12028_2023_1865_MOESM1_ESM.docx]

**Supplementary Materials Table 1 |** Multivariable logistic regression analysis of predictors for poor functional outcome at 3 months (FDP replace FPR)

| **Variables** | **OR** | **95% CI** | ***P-value*** |
| --- | --- | --- | --- |
| Age | 1.043 | 1.016-1.071 | **0.002** |
| WBC | 1.150 | 1.044-1.266 | **0.005** |
| Neutrophil | 0.952 | 0.862-1.051 | 0.328 |
| FDP | 1.059 | 1.028-1.090 | **<0.001** |
| Potassium | 0.443 | 0.247-0.796 | **0.006** |
| Lactate | 1.140 | 0.975-1.332 | 0.101 |
| mFisher score | 1.304 | 0.982-1.731 | 0.066 |
| WFNS grade | 1.276 | 1.055-1.543 | **0.012** |

WBC, white blood cells; WFNS, World Federation of Neurosurgical Society; FDP, fibrin(ogen) degradation products; OR, odds ratio; CI, confidence interval.
